# Vacuum-Assisted Closure-Assisted Reconstruction of Extensive Anterior Cervical Necrotizing Fasciitis: A Case Report

**DOI:** 10.7759/cureus.112400

**Published:** 2026-07-10

**Authors:** Deniz Hanci, Öykü İ Onaran, Bürge Dereli, Yavuz Uyar

**Affiliations:** 1 Department of Otorhinolaryngology, University of Health Sciences Prof. Dr. Cemil Taşcıoğlu City Hospital, Istanbul, TUR; 2 Department of Otorhinolaryngology, Çerkezköy State Hospital, Tekirdağ, TUR

**Keywords:** anterior cervical diskectomy, hyperbaric oxygen therapy (hbot), necrotizing fasciitis, skin grafting, vacuum-assisted closure (vac)

## Abstract

Necrotizing fasciitis (NF) is a rapidly progressive, life-threatening soft tissue infection. While the extremities and trunk are most commonly affected, the anterior cervical region represents a rare site and poses additional risks due to its proximity to vital structures such as major vessels and the airway. Prompt diagnosis and aggressive management are critical for patient survival. We report a 44-year-old previously healthy male who presented with a one-week history of worsening anterior neck infection, pain, and tissue necrosis. Laboratory tests revealed elevated acute-phase reactants, and tissue cultures grew Klebsiella pneumoniae. Imaging demonstrated subcutaneous gas and fluid collections extending from the neck into the anterior chest wall. The patient underwent seven surgical procedures, including six sessions of serial debridement and split-thickness skin grafting harvested from bilateral femoral regions. The final procedures were followed by vacuum-assisted closure (VAC) therapy to control exudate and promote graft integration. After successful graft incorporation, adjunctive hyperbaric oxygen therapy was administered. With intensive care support, pathogen-targeted antibiotic therapy, repeated surgical debridements, and meticulous wound care, the patient achieved full recovery. At the one-year follow-up, he remained in good condition without complications. Anterior cervical NF, although rare and high-risk, can be successfully treated with early diagnosis, serial surgical debridement, skin grafting, VAC therapy, and adjunctive hyperbaric oxygen therapy. This case emphasizes the importance of multidisciplinary management in treating complex cervical wounds.

## Introduction

Necrotizing fasciitis (NF) is an aggressive, rare, and life-threatening soft tissue infection with an estimated incidence of approximately four cases per 100,000 individuals [1]. It is most commonly observed in immunocompromised and elderly patients, particularly those with diabetes mellitus or HIV infection [2-4]. Among these risk factors, uncontrolled diabetes mellitus is considered the most prevalent predictor of morbidity and mortality [2]. Reported mortality rates range from 25% to 50% and increase substantially in cases requiring intensive care support [1]. Without prompt surgical intervention, outcomes are almost always fatal [5].

Although NF predominantly affects the extremities, trunk, and perineal regions, craniocervical necrotizing fasciitis (CCNF) accounts for only 1-10% of cases [5]. Within this subset, involvement of the anterior cervical region is particularly rare. When present, it poses a significant clinical challenge due to its proximity to vital structures such as the airway, major blood vessels, and cranial nerves. Odontogenic infections, maxillofacial trauma, and prior radiotherapy are among the most frequently reported etiologies of CCNF [2,6].

The rapid progression of NF is primarily driven by the spread of infection along the superficial and deep fascial planes, resulting in microvascular thrombosis, tissue ischemia, and extensive necrosis. These pathological changes impair host immune responses and reduce antibiotic penetration into infected tissues, thereby facilitating further bacterial proliferation and disease progression [7]. Hyperbaric oxygen therapy (HBOT) has been proposed as an adjunctive treatment because it increases tissue oxygen tension, enhances neutrophil-mediated bacterial killing, promotes angiogenesis and wound healing, and inhibits the growth of anaerobic bacteria. Although the routine use of HBOT remains controversial due to limited high-quality evidence, it may provide additional benefit in selected patients when combined with early aggressive surgical debridement and appropriate antimicrobial therapy [8].

Here, we present a rare case of anterior cervical NF in a previously healthy 44-year-old male who was successfully managed with serial surgical debridement, pathogen-targeted antibiotic therapy, vacuum-assisted closure (VAC) therapy, and adjunctive hyperbaric oxygen therapy. This case highlights the importance of early recognition and multidisciplinary management in achieving favorable outcomes in this high-risk condition.

## Case presentation

A 44-year-old previously healthy male presented to the emergency department with a one-week history of rapidly progressing anterior cervical infection, worsening neck pain, and visible tissue necrosis. On physical examination, extensive soft tissue destruction and necrotic areas were observed in the anterior cervical region (Figure 1), accompanied by marked swelling, erythema, and tenderness.

**Figure 1:**
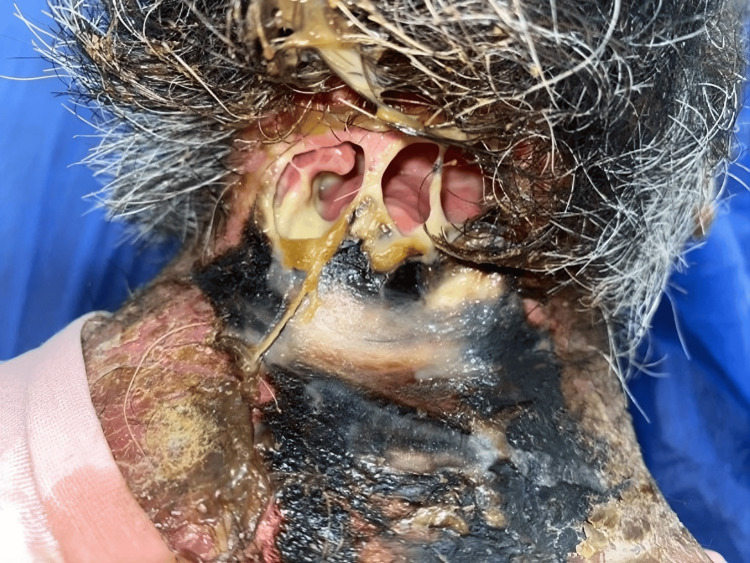
Initial presentation of the patient showing extensive anterior cervical soft tissue necrosis and tissue destruction

Initial laboratory investigations revealed markedly elevated inflammatory markers, including a C-reactive protein (CRP) level of 237 mg/L (reference range: 0-5 mg/L) and a white blood cell (WBC) count of 30.7 × 10³/µL (reference range: 4.0-10.0 × 10³/µL), indicating a severe systemic inflammatory response. During hospitalization, serial laboratory monitoring demonstrated a progressive decline in inflammatory markers following surgical debridement and targeted antimicrobial therapy, correlating with clinical improvement. To support the diagnostic evaluation, the Laboratory Risk Indicator for Necrotizing Fasciitis (LRINEC) score was calculated. The patient had markedly elevated CRP and WBC levels, while hemoglobin, sodium, creatinine, and glucose values were within normal limits. According to the LRINEC scoring system described by Wong et al. [9], the calculated LRINEC score was 6, suggesting a high suspicion for necrotizing fasciitis. Wound cultures obtained at presentation grew Klebsiella pneumoniae. The LRINEC score components included CRP 237 mg/L (4 points), WBC 30.7 × 10³/µL (2 points), hemoglobin within normal range (0 points), sodium within normal range (0 points), creatinine within normal range (0 points), and glucose within normal range (0 points), resulting in a total score of 6.

Contrast-enhanced neck CT demonstrated subcutaneous emphysema and diffuse soft tissue thickening in the bilateral lower anterior cervical regions. Thoracic CT further revealed extensive subcutaneous fluid and air collections within the anterior chest wall, with fluid accumulations measuring up to 33 mm in thickness. Although CT imaging is routinely used in diagnostic evaluation, representative radiological findings were selectively described to highlight disease extent and clinical correlation.

Based on the clinical, laboratory, and radiological findings, the patient was promptly diagnosed with necrotizing fasciitis and underwent emergent surgical debridement. Intraoperative findings revealed extensive necrosis involving the anterior cervical and upper chest wall soft tissues. Post-debridement views demonstrated deep tissue involvement and early granulation tissue formation (Figure 2).

**Figure 2:**
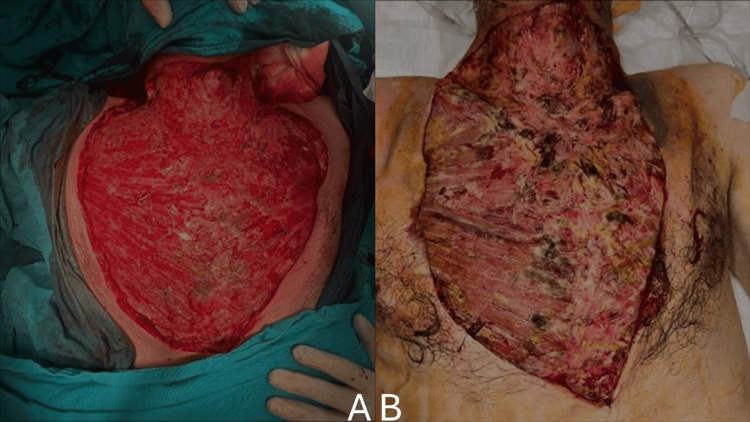
Intraoperative findings demonstrating the extent of necrosis after initial debridement A) Post-debridement view showing deep cervical and upper chest wall tissue involvement; B) Early granulation tissue formation following debridement.

Postoperatively, the patient was admitted to the intensive care unit under sedation with airway protection via endotracheal intubation. Following consultation with the infectious diseases team, intravenous meropenem and teicoplanin therapy was initiated according to tissue culture antibiotic sensitivities. Intravenous meropenem (1 g every eight hours) and teicoplanin (400 mg every 12 hours after loading dose) were administered according to culture sensitivity results.

During the subsequent clinical course, the patient underwent six additional serial debridement procedures targeting necrotic tissue in the anterior cervical and chest wall regions. In the final surgical session, split-thickness skin grafts harvested from the bilateral anterior and medial femoral regions were used to cover the extensive anterior chest wall defect. A vacuum-assisted closure (VAC) system was applied over the grafted wound to enhance graft adherence and promote granulation tissue formation (Figure 3). A portable VAC therapy device was used (V.A.C. VERAFLO™, KCI International, San Antonio, TX, USA). Negative-pressure wound therapy was applied at -125 mmHg continuous suction with dressing changes every 48-72 hours.

**Figure 3:**
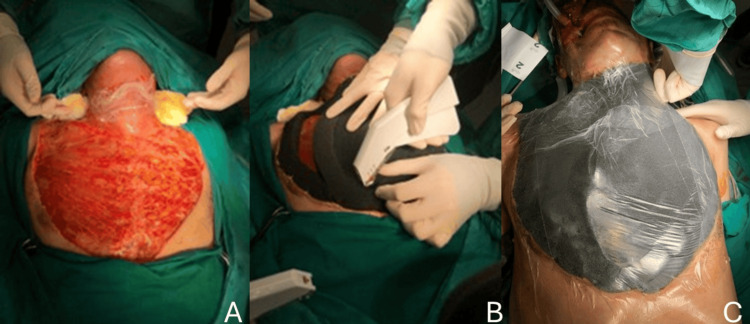
Application of vacuum-assisted closure (VAC) therapy over the debrided wound A) Demonstrating a clean wound bed prepared for negative pressure therapy; B) Placement of VAC sponge over the wound bed; C) Final VAC dressing with an airtight seal.

Following successful graft integration and granulation tissue development, the patient received adjunctive hyperbaric oxygen therapy. Hyperbaric oxygen therapy was administered at 2.5 ATA for 90 minutes per session, once daily. Progressive wound healing was observed during follow-up. At one month postoperatively, epithelialization with early scar formation was evident (Figure 4A). At the one-year follow-up, the wound demonstrated complete epithelialization with mature scar tissue formation, and the patient achieved full clinical recovery (Figure 4B). The clinical course, diagnostic work-up, and therapeutic interventions are summarized in Table 1.

**Figure 4:**
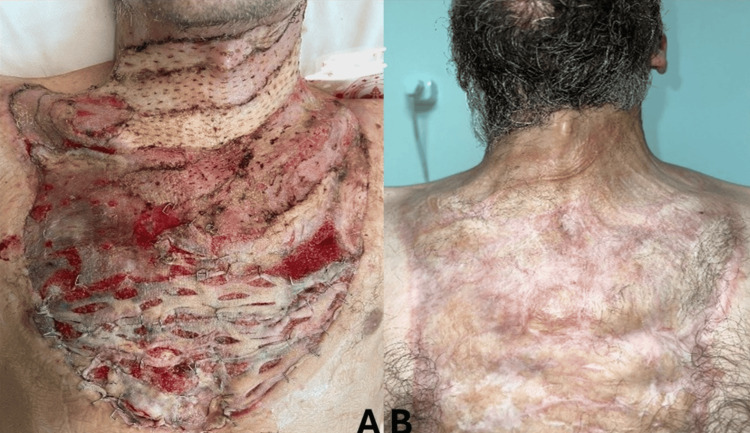
Postoperative healing and long-term follow-up A) One-month postoperative view showing progressive epithelialization and early scar formation; B) One-year follow-up demonstrating complete epithelialization with mature scar tissue formation.

**Table 1:** Clinical timeline of the patient CRP - C-reactive protein; WBC - white blood cell; LRINEC - Laboratory Risk Indicator for Necrotizing Fasciitis

Time	Clinical event
Day 0	Admission with rapidly progressive anterior cervical infection, pain, and necrosis
Day 0	Laboratory evaluation: CRP 237 mg/L, WBC 30.7 × 10³/µL, LRINEC score 6
Day 0	Contrast-enhanced CT: subcutaneous emphysema and cervicothoracic extension
Day 0	Emergency first surgical debridement performed
Days 1–10	ICU management with airway protection and IV meropenem + teicoplanin
Days 1–14	Six additional serial surgical debridements
Day ~14	Split-thickness skin grafting from bilateral femoral regions
Post-op Day ~14	Vacuum-assisted closure (VAC) therapy applied over graft
Weeks 3–4	Progressive granulation tissue formation and graft integration
After stabilization	Adjunctive hyperbaric oxygen therapy initiated
1 month	Partial epithelialization and early scar formation
1 year	Complete wound healing without recurrence or complication

## Discussion

Necrotizing fasciitis of the head and neck is a rare but rapidly progressive infection associated with high morbidity and mortality. Early recognition, prompt initiation of broad-spectrum antibiotics, and aggressive surgical debridement remain the cornerstone of management due to rapid spread along fascial planes and the risk of systemic toxicity. Surgical principles include wide exploration of affected compartments and excision of all necrotic tissue until viable bleeding margins are reached [10]. In the present case, repeated emergent debridements successfully eradicated necrotic tissue from the anterior cervical and upper chest regions, allowing for subsequent reconstructive procedures.

Craniocervical necrotizing fasciitis carries additional risk due to proximity to the airway, major vascular structures, and mediastinum. Airway compromise and descending mediastinitis are among the most severe complications and significantly increase mortality [11-12]. Although our patient did not develop these complications, early airway protection and intensive care monitoring were critical in preventing deterioration and likely contributed to the favorable outcome.

The absence of major comorbidities such as diabetes mellitus or immunosuppression may have contributed to improved prognosis. In addition, markedly elevated inflammatory markers at admission (leukocytosis and elevated CRP levels), followed by a progressive decline during hospitalization after repeated debridement and targeted antimicrobial therapy, reflected effective source control and systemic response to treatment.

Postoperative management of extensive cervical defects is challenging. Negative-pressure wound therapy (NPWT), including vacuum-assisted closure (VAC), has been widely reported as an effective adjunct in complex soft tissue infections. Previous reports have demonstrated that NPWT promotes granulation tissue formation, reduces bacterial load, and accelerates wound healing following adequate debridement [13-16]. In this case, VAC therapy applied after grafting improved exudate control, enhanced graft adherence, and stabilized the wound bed, thereby facilitating reconstruction [17].

Adjunctive hyperbaric oxygen therapy (HBO) may further improve outcomes in necrotizing soft tissue infections by enhancing tissue oxygenation, supporting leukocyte function, and promoting angiogenesis. Several case series suggest that HBO may reduce the number of required debridements and improve healing rates [18-19]. In our patient, HBO was initiated after graft integration and likely contributed to accelerated epithelialization.

Radiological imaging plays a key role in early diagnosis and surgical planning. Although contrast-enhanced CT is a valuable tool for defining disease extent and mediastinal involvement, in our case representative imaging findings were selectively described in order to focus on clinically relevant features guiding urgent surgical intervention [20].

Early differentiation of necrotizing fasciitis from severe cellulitis or deep neck infection remains challenging due to nonspecific initial presentation. However, rapid clinical deterioration, markedly elevated inflammatory markers, and the presence of gas on imaging strongly supported the diagnosis and justified immediate surgical management.

Although the Laboratory Risk Indicator for Necrotizing Fasciitis (LRINEC) score may assist in early identification, it should not delay surgical decision-making due to limited sensitivity [9,20]. In this case, a score of 6 supported high clinical suspicion.

Despite advances in management, necrotizing fasciitis remains a life-threatening condition, particularly in cases with thoracic extension or sepsis [10,12,20]. In the present case, early recognition, aggressive surgical debridement, targeted antimicrobial therapy, VAC-assisted reconstruction, and adjunctive HBO therapy collectively contributed to complete recovery.

A limitation of this report is its single-case design, which does not allow generalization of treatment efficacy. However, this case highlights the importance of a structured multidisciplinary approach in achieving favorable outcomes in extensive cervicothoracic necrotizing fasciitis.

## Conclusions

Anterior cervical necrotizing fasciitis is a rare but life-threatening condition requiring urgent diagnosis and prompt multidisciplinary management. Early surgical debridement combined with broad-spectrum antibiotics remains the cornerstone of treatment to prevent rapid disease progression and systemic complications.

In the present case, repeated surgical debridements, adjunctive vacuum-assisted closure (VAC) therapy, and hyperbaric oxygen therapy were used as part of a comprehensive multidisciplinary treatment strategy, alongside intensive critical care and targeted antimicrobial therapy. The patient achieved successful reconstruction and complete recovery following this combined approach.

This case highlights that favorable outcomes in extensive cervicocervical necrotizing fasciitis depend on early recognition and a coordinated multimodal treatment strategy rather than any single therapeutic modality.
